# (*E*)-*N*-(3,3-Di­phenyl­allyl­idene)-2-(tri­fluoro­meth­yl)aniline

**DOI:** 10.1107/S1600536813010283

**Published:** 2013-04-20

**Authors:** Byung-Yong Yu, Ji Eun Lee, Yong Seo Cho, Joo Hwan Cha, Jae Kyun Lee

**Affiliations:** a Advanced Analysis Center, Korea Institute of Science & Technology, Hwarangro 14-gil, Seongbuk-gu, Seoul 136-791, Republic of Korea; b Center for Neuro-Medicine, Brain Science Institute, Korea Institute of Science & Technology, Hwarangro 14-gil, Seongbuk-gu, Seoul 136-791, Republic of Korea

## Abstract

In the title compound, C_22_H_16_F_3_N, the C=N bond of the central imine group adopts an *E* conformation. The dihedral angles between the 2-(tri­fluoro­meth­yl)phenyl ring and the benzene rings are 9.34 (1) and 68.8 (1)°. The imine group displays a C—C—N=C torsion angle of 41.6 (3)°. In the crystal, weak C—H⋯F hydrogen bonds link the mol­ecules into chains parallel to the *b*-axis direction.

## Related literature

For the crystal structures of 2-phenyl­cinnamaldehyde derivatives studied recently our group, see: Cha *et al.* (2012 ▶); Kang *et al.* (2012 ▶).
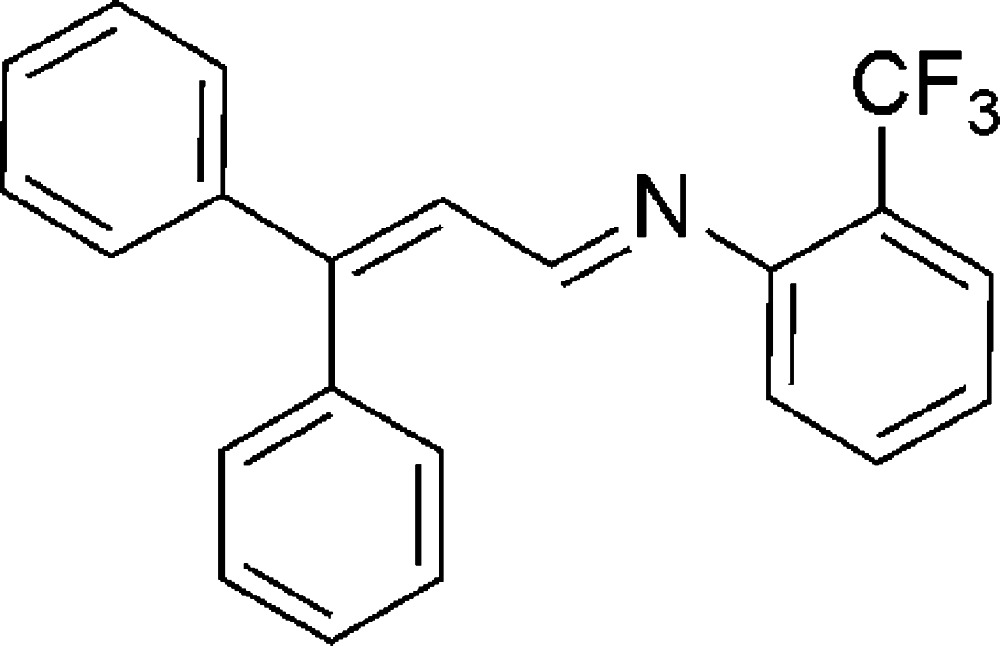


## Experimental

### 

#### Crystal data

C_22_H_16_F_3_N
*M* *_r_* = 351.37
Monoclinic, 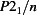

*a* = 8.6733 (8) Å
*b* = 11.8116 (9) Å
*c* = 17.6227 (15) Å
β = 95.661 (3)°
*V* = 1796.6 (3) Å^3^
*Z* = 4
Mo *K*α radiation
μ = 0.10 mm^−1^
*T* = 296 K
0.30 × 0.20 × 0.10 mm

#### Data collection

Rigaku R-AXIS RAPID diffractometer
Absorption correction: multi-scan (*ABSCOR*; Rigaku, 1995 ▶) *T*_min_ = 0.725, *T*_max_ = 0.990
16660 measured reflections
4059 independent reflections
1842 reflections with *F* ^2^ > 2σ(*F* ^2^)
*R*_int_ = 0.046

#### Refinement

*R*[*F* ^2^ > 2σ(*F* ^2^)] = 0.051
*wR*(*F* ^2^) = 0.158
*S* = 1.02
4059 reflections
243 parameters
H atoms treated by a mixture of independent and constrained refinement
Δρ_max_ = 0.20 e Å^−3^
Δρ_min_ = −0.23 e Å^−3^

### 

Data collection: *RAPID-AUTO* (Rigaku, 2006 ▶); cell refinement: *RAPID-AUTO*; data reduction: *RAPID-AUTO*; program(s) used to solve structure: *Il Milione* (Burla *et al.*, 2007 ▶); program(s) used to refine structure: *SHELXL97* (Sheldrick, 2008 ▶); molecular graphics: *CrystalStructure* (Rigaku, 2010 ▶); software used to prepare material for publication: *CrystalStructure*.

## Supplementary Material

Crystal structure: contains datablock(s) global, I. DOI: 10.1107/S1600536813010283/ff2102sup1.cif

Structure factors: contains datablock(s) I. DOI: 10.1107/S1600536813010283/ff2102Isup2.hkl

Supplementary material file. DOI: 10.1107/S1600536813010283/ff2102Isup3.cml

Additional supplementary materials:  crystallographic information; 3D view; checkCIF report

## Figures and Tables

**Table 1:** Hydrogen-bond geometry (Å, °)

*D*—H⋯*A*	*D*—H	H⋯*A*	*D*⋯*A*	*D*—H⋯*A*
C15—H15⋯F1^i^	0.93	2.52	3.392 (3)	157

Symmetry code: (i) 
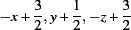
.

**Fig. 1.**
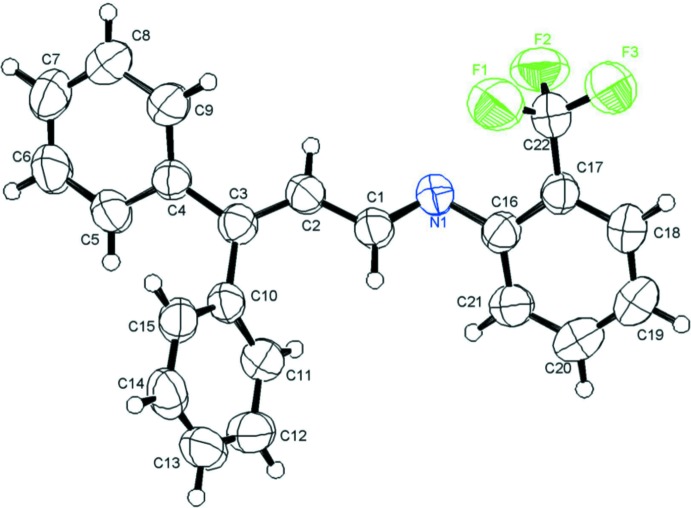
The molecular structure of the title compound showing the atomic numbering and 50% probability displacement ellipsoid.

**Fig. 2.**
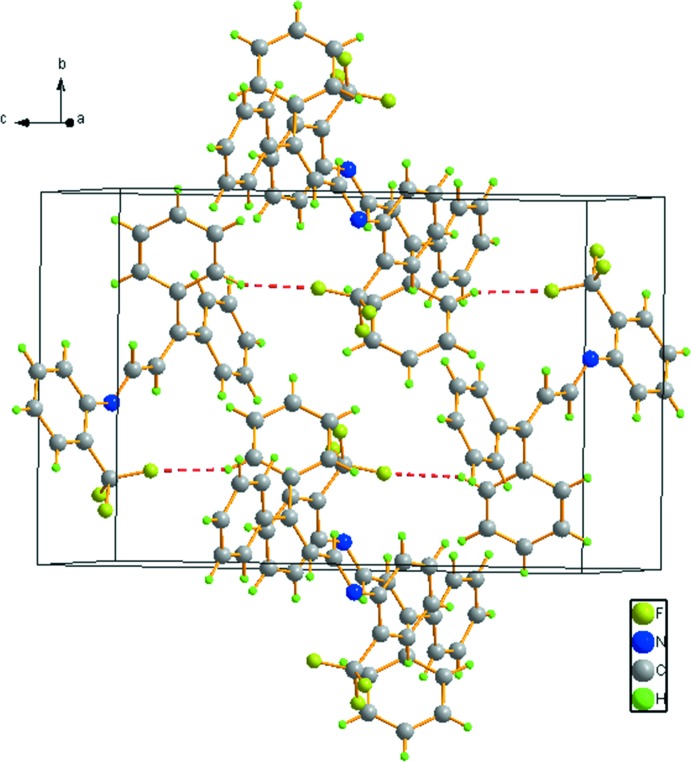
The crystal packing of the title compound. The H atoms not involved in the intermolecular interactions (dotted lines) have been omitted for clarity.

## supplementary crystallographic information

### Comment

As part of our ongoing study of the substituent effect on the solid state structures of 2-phenylcinnamaldehyde derivatives Cha *et al.*, (2012); Kang *et al.*, (2012).

In the title compound(Fig. 1), C_22_H_16_N_1_F_3_, the C=N bond of the central imine group adopts an *E* conformation. The dihedral angles between the mean planes of the central 2-trifluoromethylphenyl ring on the one hand and phenyl rings are (C4/C5/C6/C7/C8/C9) 9.344 (1)° and (C11/C11/C12/C13/C14/C15) 68.8 (1)°, respectively. The imine group displays a torsion angle [C21—C16—N1=C1 = 41.6 (3)°]. In the crystal, weak intermolecular C—H···F hydrogen bonds (Table 1) link molecules into chains parallel to the *b* axis (Fig. 2).

### Experimental

To a solution of 2-trifluoromethyl aniline (4.0 mmol) in ethanol (20 ml) was treated with equimolar quantities of substituted 2-phenylcinnamaldehydes. The mixture was refluxed for 24 h, and the progress of reaction was monitored by TLC. Upen completion,the solvent was removed under reduced pressure. The residue was purified by flash column chromatography to afford the title compound in 73% yield. Recrystallization from ethanol gave crystals suitable for X-ray analysis.

### Refinement

All hydrogen atoms were positioned geometrically (C—H = 0.93 Å), and refined using a riding model, with *U*_iso_(H) = 1.2 *U*_eq_(C).

### Crystal data

**Table:**

C_22_H_16_F_3_N	*F*(000) = 728.00
*M**_r_* = 351.37	*D*_x_ = 1.299 Mg m^−^^3^
Monoclinic, *P*2_1_/*n*	Mo *K*α radiation, λ = 0.71075 Å
Hall symbol: -P 2yn	Cell parameters from 8333 reflections
*a* = 8.6733 (8) Å	θ = 3.1–27.5°
*b* = 11.8116 (9) Å	µ = 0.10 mm^−^^1^
*c* = 17.6227 (15) Å	*T* = 296 K
β = 95.661 (3)°	Chunk, yellow
*V* = 1796.6 (3) Å^3^	0.30 × 0.20 × 0.10 mm
*Z* = 4	

### Data collection

**Table:**

Rigaku R-AXIS RAPID diffractometer	1842 reflections with *F*^2^ > 2σ(*F*^2^)
Detector resolution: 10.000 pixels mm^-1^	*R*_int_ = 0.046
ω scans	θ_max_ = 27.5°
Absorption correction: multi-scan (*ABSCOR*; Rigaku, 1995)	*h* = −11→11
*T*_min_ = 0.725, *T*_max_ = 0.990	*k* = −14→14
16660 measured reflections	*l* = −22→21
4059 independent reflections	

### Refinement

**Table:**

Refinement on *F*^2^	Secondary atom site location: difference Fourier map
*R*[*F*^2^ > 2σ(*F*^2^)] = 0.051	Hydrogen site location: inferred from neighbouring sites
*wR*(*F*^2^) = 0.158	H atoms treated by a mixture of independent and constrained refinement
*S* = 1.02	*w* = 1/[σ^2^(*F*_o_^2^) + (0.0699*P*)^2^ + 0.1152*P*] where *P* = (*F*_o_^2^ + 2*F*_c_^2^)/3
4059 reflections	(Δ/σ)_max_ < 0.001
243 parameters	Δρ_max_ = 0.20 e Å^−^^3^
0 restraints	Δρ_min_ = −0.23 e Å^−^^3^
Primary atom site location: structure-invariant direct methods	

### Special details

**Table:**

Geometry. All e.s.d.'s (except the e.s.d. in the dihedral angle between two l.s. planes) are estimated using the full covariance matrix. The cell e.s.d.'s are taken into account individually in the estimation of e.s.d.'s in distances, angles and torsion angles; correlations between e.s.d.'s in cell parameters are only used when they are defined by crystal symmetry. An approximate (isotropic) treatment of cell e.s.d.'s is used for estimating e.s.d.'s involving l.s. planes.Y
Refinement. Refinement was performed using all reflections. The weighted *R*-factor (*wR*) and goodness of fit (*S*) are based on *F*^2^. *R*-factor (gt) are based on *F*. The threshold expression of *F*^2^ > 2.0 σ(*F*^2^) is used only for calculating *R*-factor (gt).

### Fractional atomic coordinates and isotropic or equivalent isotropic displacement parameters (Å^2^)

**Table:**

	*x*	*y*	*z*	*U*_iso_*/*U*_eq_	
F1	0.6398 (3)	−0.25532 (14)	0.58228 (9)	0.1171 (7)	
F2	0.8032 (2)	−0.32028 (13)	0.51242 (10)	0.1025 (6)	
F3	0.5690 (2)	−0.36508 (13)	0.49031 (10)	0.1144 (7)	
N1	0.8301 (2)	−0.07184 (14)	0.53713 (10)	0.0613 (5)	
C1	0.8428 (3)	0.01876 (18)	0.57689 (12)	0.0574 (6)	
C2	0.9652 (3)	0.03036 (19)	0.63772 (13)	0.0592 (6)	
C3	0.9969 (3)	0.12242 (16)	0.68171 (11)	0.0539 (6)	
C4	1.1215 (3)	0.11851 (16)	0.74583 (12)	0.0564 (6)	
C5	1.2131 (3)	0.21263 (19)	0.76457 (14)	0.0721 (7)	
C6	1.3343 (4)	0.2068 (3)	0.82101 (16)	0.0859 (8)	
C7	1.3664 (4)	0.1084 (3)	0.86058 (16)	0.0892 (9)	
C8	1.2762 (4)	0.0149 (3)	0.84402 (15)	0.0873 (8)	
C9	1.1545 (3)	0.0196 (2)	0.78744 (13)	0.0721 (7)	
C10	0.9134 (3)	0.23139 (16)	0.66760 (12)	0.0549 (6)	
C11	0.9006 (3)	0.28326 (18)	0.59672 (13)	0.0660 (7)	
C12	0.8215 (4)	0.3845 (2)	0.58486 (16)	0.0815 (8)	
C13	0.7547 (4)	0.4348 (2)	0.6434 (2)	0.0874 (9)	
C14	0.7667 (3)	0.3857 (3)	0.71360 (17)	0.0812 (8)	
C15	0.8459 (3)	0.28419 (19)	0.72649 (13)	0.0664 (6)	
C16	0.7127 (3)	−0.07673 (18)	0.47539 (12)	0.0571 (6)	
C17	0.6337 (3)	−0.17848 (17)	0.45946 (12)	0.0569 (6)	
C18	0.5267 (3)	−0.1866 (2)	0.39554 (13)	0.0695 (7)	
C19	0.4984 (3)	−0.0951 (3)	0.34748 (14)	0.0772 (7)	
C20	0.5743 (3)	0.0049 (3)	0.36362 (14)	0.0773 (7)	
C21	0.6804 (3)	0.01425 (19)	0.42670 (13)	0.0680 (7)	
C22	0.6603 (4)	−0.2777 (2)	0.51082 (14)	0.0707 (7)	
H5	1.1924	0.2804	0.7387	0.0865*	
H6	1.3953	0.2704	0.8325	0.1031*	
H7	1.4489	0.1051	0.8985	0.1070*	
H8	1.2969	−0.0520	0.8710	0.1048*	
H9	1.0935	−0.0443	0.7769	0.0866*	
H11	0.9458	0.2496	0.5566	0.0791*	
H12	0.8138	0.4184	0.5370	0.0978*	
H13	0.7011	0.5025	0.6352	0.1049*	
H14	0.7215	0.4204	0.7533	0.0975*	
H15	0.8537	0.2515	0.7747	0.0797*	
H18	0.4738	−0.2541	0.3851	0.0834*	
H19	0.4280	−0.1014	0.3043	0.0927*	
H20	0.5541	0.0669	0.3317	0.0928*	
H21	0.7312	0.0827	0.4369	0.0816*	
H1	0.765 (3)	0.0828 (19)	0.5653 (12)	0.072 (7)*	
H2	1.023 (3)	−0.035 (2)	0.6462 (12)	0.075 (8)*	

### Atomic displacement parameters (Å^2^)

**Table:**

	*U*^11^	*U*^22^	*U*^33^	*U*^12^	*U*^13^	*U*^23^
F1	0.193 (2)	0.0927 (11)	0.0683 (11)	0.0047 (12)	0.0267 (11)	0.0112 (8)
F2	0.0944 (13)	0.0818 (10)	0.1279 (14)	0.0183 (9)	−0.0063 (10)	0.0169 (9)
F3	0.1240 (15)	0.0775 (10)	0.1332 (15)	−0.0340 (10)	−0.0301 (12)	0.0114 (9)
N1	0.0636 (13)	0.0547 (10)	0.0633 (12)	−0.0003 (9)	−0.0048 (9)	−0.0074 (9)
C1	0.0548 (14)	0.0545 (13)	0.0620 (14)	−0.0007 (11)	0.0018 (11)	−0.0049 (10)
C2	0.0575 (15)	0.0519 (13)	0.0669 (15)	0.0027 (11)	−0.0005 (11)	−0.0056 (11)
C3	0.0507 (13)	0.0534 (12)	0.0573 (13)	−0.0021 (10)	0.0043 (10)	−0.0015 (10)
C4	0.0534 (13)	0.0561 (12)	0.0591 (13)	0.0033 (10)	0.0022 (10)	−0.0052 (10)
C5	0.0688 (16)	0.0559 (13)	0.0880 (18)	−0.0007 (12)	−0.0110 (14)	−0.0096 (12)
C6	0.0771 (19)	0.0716 (16)	0.103 (3)	−0.0068 (14)	−0.0211 (16)	−0.0190 (15)
C7	0.081 (2)	0.099 (2)	0.0819 (19)	0.0070 (17)	−0.0241 (15)	−0.0146 (16)
C8	0.092 (3)	0.0839 (18)	0.0800 (19)	0.0046 (16)	−0.0203 (16)	0.0108 (14)
C9	0.0728 (17)	0.0673 (15)	0.0730 (16)	−0.0046 (12)	−0.0094 (13)	0.0058 (12)
C10	0.0524 (13)	0.0519 (12)	0.0591 (14)	−0.0013 (10)	−0.0006 (10)	−0.0062 (10)
C11	0.0695 (16)	0.0606 (14)	0.0674 (16)	−0.0030 (12)	0.0049 (12)	0.0013 (11)
C12	0.0862 (19)	0.0659 (16)	0.089 (2)	−0.0006 (14)	−0.0073 (15)	0.0148 (14)
C13	0.079 (2)	0.0574 (15)	0.121 (3)	0.0092 (14)	−0.0190 (17)	−0.0074 (16)
C14	0.0677 (17)	0.0790 (17)	0.094 (2)	0.0135 (14)	−0.0072 (14)	−0.0337 (15)
C15	0.0635 (15)	0.0712 (15)	0.0626 (15)	0.0081 (12)	−0.0036 (12)	−0.0143 (11)
C16	0.0555 (14)	0.0605 (13)	0.0544 (13)	0.0055 (11)	0.0009 (11)	−0.0040 (10)
C17	0.0582 (14)	0.0591 (13)	0.0528 (13)	0.0015 (11)	0.0027 (11)	−0.0055 (10)
C18	0.0652 (16)	0.0785 (16)	0.0636 (15)	−0.0063 (13)	−0.0003 (12)	−0.0088 (12)
C19	0.0666 (17)	0.100 (2)	0.0626 (16)	0.0043 (15)	−0.0056 (12)	0.0031 (14)
C20	0.0722 (18)	0.0903 (18)	0.0687 (16)	0.0106 (15)	0.0025 (14)	0.0171 (13)
C21	0.0690 (16)	0.0639 (14)	0.0702 (16)	0.0019 (12)	0.0027 (13)	0.0042 (12)
C22	0.0763 (18)	0.0647 (15)	0.0688 (17)	−0.0060 (14)	−0.0043 (13)	−0.0077 (12)

### Geometric parameters (Å, º)

**Table:**

F1—C22	1.316 (3)	C16—C21	1.386 (3)
F2—C22	1.335 (4)	C17—C18	1.390 (3)
F3—C22	1.330 (3)	C17—C22	1.484 (4)
N1—C1	1.278 (3)	C18—C19	1.380 (4)
N1—C16	1.416 (3)	C19—C20	1.368 (4)
C1—C2	1.439 (3)	C20—C21	1.376 (4)
C2—C3	1.348 (3)	C1—H1	1.02 (3)
C3—C4	1.485 (3)	C2—H2	0.92 (3)
C3—C10	1.486 (3)	C5—H5	0.930
C4—C5	1.387 (3)	C6—H6	0.930
C4—C9	1.394 (3)	C7—H7	0.930
C5—C6	1.376 (4)	C8—H8	0.930
C6—C7	1.370 (4)	C9—H9	0.930
C7—C8	1.368 (4)	C11—H11	0.930
C8—C9	1.379 (4)	C12—H12	0.930
C10—C11	1.386 (3)	C13—H13	0.930
C10—C15	1.389 (4)	C14—H14	0.930
C11—C12	1.384 (4)	C15—H15	0.930
C12—C13	1.368 (5)	C18—H18	0.930
C13—C14	1.361 (5)	C19—H19	0.930
C14—C15	1.389 (4)	C20—H20	0.930
C16—C17	1.398 (3)	C21—H21	0.930
			
F1···N1	2.883 (3)	C11···H8^vi^	3.2704
F1···C16	2.938 (3)	C11···H18^v^	3.3122
F1···C18	3.437 (3)	C12···H7^vi^	3.2754
F2···N1	2.972 (3)	C12···H7^xi^	3.5695
F2···C16	3.036 (3)	C12···H8^vi^	3.4076
F2···C18	3.393 (3)	C12···H18^v^	3.0784
F3···C18	2.692 (3)	C13···H7^vi^	3.4022
N1···C22	2.856 (3)	C13···H9^vii^	3.4634
C1···C10	3.008 (3)	C13···H18^v^	2.9214
C1···C11	3.178 (3)	C14···H9^vii^	3.2524
C1···C17	3.501 (3)	C14···H18^v^	3.0123
C1···C21	2.873 (3)	C14···H19^x^	3.2502
C2···C9	2.970 (4)	C14···H20^x^	3.1361
C2···C11	3.111 (3)	C15···H18^v^	3.2565
C2···C15	3.581 (4)	C15···H19^v^	3.2166
C4···C7	2.787 (4)	C15···H20^x^	3.0218
C4···C15	3.082 (3)	C16···H2^iii^	3.54 (3)
C5···C8	2.751 (4)	C18···H1^v^	2.95 (3)
C5···C10	2.975 (4)	C19···H14^xi^	3.4634
C5···C15	3.300 (4)	C19···H1^v^	2.88 (3)
C6···C9	2.736 (4)	C20···H5^xi^	3.5740
C10···C13	2.781 (4)	C20···H14^xi^	3.5708
C11···C14	2.744 (4)	C20···H1^v^	3.47 (3)
C12···C15	2.752 (4)	C20···H2^iii^	3.53 (3)
C16···C19	2.785 (4)	C21···H2^iii^	3.00 (3)
C17···C20	2.763 (4)	C22···H8^ix^	3.4788
C18···C21	2.750 (4)	C22···H13^viii^	3.3938
F1···C15^i^	3.392 (3)	H5···C8^vi^	3.1537
F2···C7^ii^	3.559 (4)	H5···C9^vi^	3.1744
F2···C11^iii^	3.386 (3)	H5···C20^x^	3.5740
F3···F3^iv^	3.433 (3)	H5···H8^vi^	2.7740
F3···C12^v^	3.521 (4)	H5···H9^vi^	2.8126
F3···C13^v^	3.579 (4)	H5···H20^x^	2.7867
C1···C19^v^	3.482 (4)	H6···C2^vi^	3.3227
C2···C21^iii^	3.421 (4)	H6···H9^vi^	2.9235
C7···F2^vi^	3.559 (4)	H6···H19^iii^	3.5879
C11···F2^iii^	3.386 (3)	H6···H20^x^	3.5274
C12···F3^v^	3.521 (4)	H6···H21^x^	2.9893
C13···F3^v^	3.579 (4)	H6···H2^vi^	2.4273
C15···F1^vii^	3.392 (3)	H7···F2^vi^	2.6835
C19···C1^v^	3.482 (4)	H7···C12^ii^	3.2754
C21···C2^iii^	3.421 (4)	H7···C12^x^	3.5695
F2···H18	3.5415	H7···C13^ii^	3.4022
F3···H18	2.3524	H7···H11^x^	3.2752
N1···H21	2.6255	H7···H12^ii^	3.1520
N1···H2	2.46 (2)	H7···H12^x^	2.8240
C1···H11	2.9026	H7···H13^ii^	3.3753
C1···H21	2.6696	H8···F2^xii^	2.9097
C2···H9	2.7372	H8···F3^xii^	3.1798
C2···H11	2.9549	H8···C11^ii^	3.2704
C3···H5	2.6514	H8···C12^ii^	3.4076
C3···H9	2.6674	H8···C22^xii^	3.4788
C3···H11	2.6685	H8···H5^ii^	2.7740
C3···H15	2.6371	H8···H11^ii^	3.3964
C3···H1	2.77 (2)	H8···H12^x^	3.3155
C4···H6	3.2368	H9···C5^ii^	3.4397
C4···H8	3.2516	H9···C6^ii^	3.4967
C4···H15	2.8899	H9···C13^i^	3.4634
C4···H2	2.60 (3)	H9···C14^i^	3.2524
C5···H7	3.2263	H9···H5^ii^	2.8126
C5···H9	3.2213	H9···H6^ii^	2.9235
C5···H15	3.1731	H9···H13^i^	3.1647
C6···H8	3.2062	H9···H14^i^	2.7633
C7···H5	3.2218	H9···H18^xii^	3.2826
C7···H9	3.2154	H11···F2^iii^	2.7276
C8···H6	3.2039	H11···N1^iii^	3.3985
C9···H5	3.2236	H11···H7^xi^	3.2752
C9···H7	3.2220	H11···H8^vi^	3.3964
C9···H2	2.71 (2)	H12···F2^xiii^	3.1174
C10···H5	2.6762	H12···F3^xiii^	3.3739
C10···H12	3.2448	H12···F3^v^	3.3667
C10···H14	3.2449	H12···C7^xi^	3.2020
C10···H1	2.75 (3)	H12···C8^xi^	3.4750
C10···H2	3.32 (3)	H12···H7^vi^	3.1520
C11···H5	3.3787	H12···H7^xi^	2.8240
C11···H13	3.2243	H12···H8^xi^	3.3155
C11···H15	3.2246	H12···H18^v^	3.5413
C11···H1	2.68 (3)	H13···F1^xiii^	3.0388
C12···H14	3.2015	H13···F2^xiii^	3.1975
C13···H11	3.2197	H13···F3^xiii^	3.1146
C13···H15	3.2232	H13···F3^v^	3.4632
C14···H12	3.2018	H13···C9^vii^	3.4987
C15···H5	2.9921	H13···C22^xiii^	3.3938
C15···H11	3.2234	H13···H7^vi^	3.3753
C15···H13	3.2278	H13···H9^vii^	3.1647
C16···H18	3.2510	H13···H15^vii^	3.3971
C16···H20	3.2397	H13···H18^v^	3.3058
C16···H1	2.47 (3)	H14···C1^vii^	3.3088
C17···H19	3.2440	H14···C2^vii^	2.9347
C17···H21	3.2342	H14···C3^vii^	3.3205
C18···H20	3.2155	H14···C9^vii^	3.4734
C19···H21	3.2162	H14···C19^x^	3.4634
C20···H18	3.2138	H14···C20^x^	3.5708
C21···H19	3.2214	H14···H9^vii^	2.7633
C21···H1	2.61 (2)	H14···H18^v^	3.4433
C22···H18	2.6261	H14···H19^x^	2.8757
H5···H6	2.2951	H14···H20^x^	3.0769
H5···H15	3.0848	H14···H2^vii^	2.9439
H6···H7	2.2967	H15···F1^vii^	2.5164
H7···H8	2.3005	H15···H13^i^	3.3971
H8···H9	2.3004	H15···H19^v^	3.2222
H9···H2	2.3263	H15···H20^x^	2.8785
H11···H12	2.3075	H18···C10^v^	3.4054
H11···H21	3.3212	H18···C11^v^	3.3122
H11···H1	2.5338	H18···C12^v^	3.0784
H12···H13	2.2964	H18···C13^v^	2.9214
H13···H14	2.2869	H18···C14^v^	3.0123
H14···H15	2.3135	H18···C15^v^	3.2565
H18···H19	2.3084	H18···H9^ix^	3.2826
H19···H20	2.2971	H18···H12^v^	3.5413
H20···H21	2.2948	H18···H13^v^	3.3058
H21···H1	2.2529	H18···H14^v^	3.4433
H1···H2	2.89 (3)	H18···H1^v^	3.0812
F1···H13^viii^	3.0388	H19···C1^v^	3.4381
F1···H15^i^	2.5164	H19···C6^iii^	3.4020
F1···H20^v^	3.2530	H19···C7^iii^	3.5573
F2···H7^ii^	2.6835	H19···C10^v^	3.4148
F2···H8^ix^	2.9097	H19···C14^xi^	3.2502
F2···H11^iii^	2.7276	H19···C15^v^	3.2166
F2···H12^viii^	3.1174	H19···H6^iii^	3.5879
F2···H13^viii^	3.1975	H19···H14^xi^	2.8757
F3···H8^ix^	3.1798	H19···H15^v^	3.2222
F3···H12^viii^	3.3739	H19···H1^v^	2.9802
F3···H12^v^	3.3667	H20···F1^v^	3.2530
F3···H13^viii^	3.1146	H20···C5^xi^	3.2257
F3···H13^v^	3.4632	H20···C9^iii^	3.5897
N1···H11^iii^	3.3985	H20···C14^xi^	3.1361
C1···H14^i^	3.3088	H20···C15^xi^	3.0218
C1···H19^v^	3.4381	H20···H5^xi^	2.7867
C2···H6^ii^	3.3227	H20···H6^xi^	3.5274
C2···H14^i^	2.9347	H20···H14^xi^	3.0769
C2···H21^iii^	3.3361	H20···H15^xi^	2.8785
C3···H14^i^	3.3205	H21···C2^iii^	3.3361
C5···H9^vi^	3.4397	H21···C6^xi^	3.3927
C5···H20^x^	3.2257	H21···H6^xi^	2.9893
C6···H9^vi^	3.4967	H21···H2^iii^	2.7602
C6···H19^iii^	3.4020	H1···C18^v^	2.95 (3)
C6···H21^x^	3.3927	H1···C19^v^	2.88 (3)
C6···H2^vi^	3.32 (3)	H1···C20^v^	3.47 (3)
C7···H12^x^	3.2020	H1···H18^v^	3.0812
C7···H19^iii^	3.5573	H1···H19^v^	2.9802
C8···H5^ii^	3.1537	H2···C6^ii^	3.32 (3)
C8···H12^x^	3.4750	H2···C16^iii^	3.54 (3)
C9···H5^ii^	3.1744	H2···C20^iii^	3.53 (3)
C9···H13^i^	3.4987	H2···C21^iii^	3.00 (3)
C9···H14^i^	3.4734	H2···H6^ii^	2.4273
C9···H20^iii^	3.5897	H2···H14^i^	2.9439
C10···H18^v^	3.4054	H2···H21^iii^	2.7602
C10···H19^v^	3.4148		
			
C1—N1—C16	118.15 (18)	F2—C22—F3	103.9 (2)
N1—C1—C2	120.4 (2)	F2—C22—C17	113.5 (3)
C1—C2—C3	126.8 (2)	F3—C22—C17	113.2 (2)
C2—C3—C4	120.31 (19)	N1—C1—H1	119.4 (12)
C2—C3—C10	122.27 (19)	C2—C1—H1	120.3 (12)
C4—C3—C10	117.41 (17)	C1—C2—H2	113.1 (14)
C3—C4—C5	120.86 (19)	C3—C2—H2	120.1 (14)
C3—C4—C9	121.52 (19)	C4—C5—H5	119.643
C5—C4—C9	117.6 (2)	C6—C5—H5	119.648
C4—C5—C6	120.7 (3)	C5—C6—H6	119.575
C5—C6—C7	120.9 (3)	C7—C6—H6	119.568
C6—C7—C8	119.6 (3)	C6—C7—H7	120.214
C7—C8—C9	120.1 (3)	C8—C7—H7	120.218
C4—C9—C8	121.1 (3)	C7—C8—H8	119.943
C3—C10—C11	121.9 (2)	C9—C8—H8	119.939
C3—C10—C15	119.90 (19)	C4—C9—H9	119.432
C11—C10—C15	118.2 (2)	C8—C9—H9	119.422
C10—C11—C12	120.8 (3)	C10—C11—H11	119.587
C11—C12—C13	120.1 (3)	C12—C11—H11	119.590
C12—C13—C14	120.0 (3)	C11—C12—H12	119.939
C13—C14—C15	120.6 (3)	C13—C12—H12	119.934
C10—C15—C14	120.2 (3)	C12—C13—H13	119.980
N1—C16—C17	119.37 (19)	C14—C13—H13	119.993
N1—C16—C21	122.02 (19)	C13—C14—H14	119.706
C17—C16—C21	118.46 (19)	C15—C14—H14	119.723
C16—C17—C18	119.8 (2)	C10—C15—H15	119.882
C16—C17—C22	120.79 (19)	C14—C15—H15	119.890
C18—C17—C22	119.4 (2)	C17—C18—H18	119.747
C17—C18—C19	120.5 (3)	C19—C18—H18	119.754
C18—C19—C20	119.7 (3)	C18—C19—H19	120.153
C19—C20—C21	120.5 (3)	C20—C19—H19	120.159
C16—C21—C20	121.1 (3)	C19—C20—H20	119.752
F1—C22—F2	105.6 (2)	C21—C20—H20	119.751
F1—C22—F3	106.4 (3)	C16—C21—H21	119.469
F1—C22—C17	113.4 (2)	C20—C21—H21	119.473
			
C1—N1—C16—C17	−142.8 (2)	C11—C10—C15—C14	0.6 (3)
C1—N1—C16—C21	41.6 (3)	C15—C10—C11—C12	−0.5 (3)
C16—N1—C1—C2	−176.75 (17)	C10—C11—C12—C13	0.0 (4)
N1—C1—C2—C3	176.6 (2)	C11—C12—C13—C14	0.4 (4)
C1—C2—C3—C4	175.9 (2)	C12—C13—C14—C15	−0.3 (4)
C1—C2—C3—C10	−5.5 (4)	C13—C14—C15—C10	−0.2 (4)
C2—C3—C4—C5	144.6 (2)	N1—C16—C17—C18	−175.09 (18)
C2—C3—C4—C9	−33.0 (3)	N1—C16—C17—C22	6.1 (3)
C2—C3—C10—C11	−52.5 (3)	N1—C16—C21—C20	174.87 (19)
C2—C3—C10—C15	127.7 (3)	C17—C16—C21—C20	−0.7 (4)
C4—C3—C10—C11	126.1 (2)	C21—C16—C17—C18	0.6 (4)
C4—C3—C10—C15	−53.7 (3)	C21—C16—C17—C22	−178.24 (19)
C10—C3—C4—C5	−34.0 (3)	C16—C17—C18—C19	0.3 (4)
C10—C3—C4—C9	148.46 (18)	C16—C17—C22—F1	54.5 (3)
C3—C4—C5—C6	−176.09 (19)	C16—C17—C22—F2	−66.0 (3)
C3—C4—C9—C8	176.22 (19)	C16—C17—C22—F3	175.9 (2)
C5—C4—C9—C8	−1.4 (4)	C18—C17—C22—F1	−124.4 (3)
C9—C4—C5—C6	1.6 (4)	C18—C17—C22—F2	115.1 (3)
C4—C5—C6—C7	−0.7 (4)	C18—C17—C22—F3	−3.0 (4)
C5—C6—C7—C8	−0.3 (5)	C22—C17—C18—C19	179.2 (2)
C6—C7—C8—C9	0.5 (5)	C17—C18—C19—C20	−1.1 (4)
C7—C8—C9—C4	0.4 (4)	C18—C19—C20—C21	1.0 (4)
C3—C10—C11—C12	179.65 (17)	C19—C20—C21—C16	−0.1 (4)
C3—C10—C15—C14	−179.55 (17)		

Symmetry codes: (i) −*x*+3/2, *y*−1/2, −*z*+3/2; (ii) −*x*+5/2, *y*−1/2, −*z*+3/2; (iii) −*x*+2, −*y*, −*z*+1; (iv) −*x*+1, −*y*−1, −*z*+1; (v) −*x*+1, −*y*, −*z*+1; (vi) −*x*+5/2, *y*+1/2, −*z*+3/2; (vii) −*x*+3/2, *y*+1/2, −*z*+3/2; (viii) *x*, *y*−1, *z*; (ix) *x*−1/2, −*y*−1/2, *z*−1/2; (x) *x*+1/2, −*y*+1/2, *z*+1/2; (xi) *x*−1/2, −*y*+1/2, *z*−1/2; (xii) *x*+1/2, −*y*−1/2, *z*+1/2; (xiii) *x*, *y*+1, *z*.

### Hydrogen-bond geometry (Å, º)

**Table:**

*D*—H···*A*	*D*—H	H···*A*	*D*···*A*	*D*—H···*A*
C15—H15···F1^vii^	0.93	2.52	3.392 (3)	157

Symmetry code: (vii) −*x*+3/2, *y*+1/2, −*z*+3/2.

## References

[bb1] Burla, M. C., Caliandro, R., Camalli, M., Carrozzini, B., Cascarano, G. L., De Caro, L., Giacovazzo, C., Polidori, G., Siliqi, D. & Spagna, R. (2007). *J. Appl. Cryst.* **40**, 609–613.

[bb2] Cha, J. H., Kang, Y. K., Cho, Y. S., Lee, J. K. & Woo, J. C. (2012). *Acta Cryst.* E**68**, o3030. 10.1107/S1600536812040354 PMC3470384 23125797

[bb3] Kang, Y. K., Cho, Y. S., Lee, J. K., Yu, B.-Y. & Cha, J. H. (2012). *Acta Cryst.* E**68**, o3031. 10.1107/S1600536812040391 PMC3470385 23125798

[bb4] Rigaku (1995). *ABSCOR* Rigaku Corporation, Tokyo, Japan.

[bb5] Rigaku (2006). *RAPID-AUTO* Rigaku Corporation, Tokyo, Japan.

[bb6] Rigaku (2010). *CrystalStructure* Rigaku Corporation, Tokyo, Japan.

[bb7] Sheldrick, G. M. (2008). *Acta Cryst.* A**64**, 112–122. 10.1107/S0108767307043930 18156677

